# T cell immunoglobulin and mucin domain-containing protein 3 is highly expressed in patients with acute decompensated heart failure and predicts mid-term prognosis

**DOI:** 10.3389/fcvm.2022.933532

**Published:** 2022-09-15

**Authors:** Xin Meng, Guofang Xia, Lili Zhang, Congfeng Xu, Zhong Chen

**Affiliations:** Department of Cardiology, The Affiliated Sixth People’s Hospital, Shanghai Jiao Tong University School of Medicine, Shanghai, China

**Keywords:** acute decompensated heart failure, Tim-3, T-lymphocytes, prognosis, heart failure

## Abstract

**Background and aims:**

T cell immunoglobulin and mucin domain-containing protein 3 (Tim-3) is mainly expressed by immune cells and plays an immunomodulatory role in cardiovascular disease. However, the prognostic value of Tim-3 in acute decompensated heart failure (ADHF) is unclear. This study aimed to investigate the expression profile of Tim-3 on CD4^+^ and CD8^+^ T cells in patients with ADHF and its impact on their prognosis.

**Methods:**

In this prospective study, 84 patients who were hospitalized with ADHF and 83 patients without heart failure were enrolled. Main clinical data were collected during patient visits. The Tim-3 expression on CD4^+^ and CD8^+^ T cells in peripheral blood samples was assayed by flow cytometry. Long-term prognosis of the patients with ADHF was evaluated by major adverse cardiac and cerebrovascular events (MACCE) over a 12-month follow-up period.

**Results:**

We found that the Tim-3 expression on CD4^+^ T cells [2.08% (1.15–2.67%) vs. 0.88% (0.56–1.39%), *p* < 0.001] and CD8^+^ T cells [3.81% (2.24–6.03%) vs. 1.36% (0.76–3.00%), *p* < 0.001] in ADHF group were significantly increased vs. the non-ADHF group. Logistic analysis revealed that high levels of Tim-3 expressed on CD4^+^ and CD8^+^ T cells were independent risk factors of ADHF (OR: 2.76; 95% CI: 1.34–5.65, *p* = 0.006; OR: 2.58; 95% CI: 1.26–5.31, *p* = 0.010, respectively). ROC curve analysis showed that the high level of Tim-3 on CD4^+^ or CD8^+^ T cells as a biomarker has predictive performance for ADHF (AUC: 0.75; 95% CI: 0.68–0.83; AUC: 0.78, 95% CI: 0.71–0.85, respectively). During a median follow-up of 12 months, the Cox regression analysis revealed that higher Tim-3 on CD4^+^ and CD8^+^ T cells were strongly associated with increased risks of MACCE within 12 months after ADHF (HR: 2.613; 95% CI: 1.11–6.13, *p* = 0.027; HR: 2.762, 95% CI: 1.15–6.63, *p* = 0.023; respectively).

**Conclusion:**

Our research indicated that the expression level of Tim-3 on CD4^+^ and CD8^+^ T cells, elevated in patients with ADHF, was an independent predictor of MACCE within 12 months after ADHF. It suggests a potential immunoregulatory role of Tim-3 signaling system in the mechanism of ADHF.

## Introduction

Acute decompensated heart failure (ADHF) has become a significant medical, social, and economic problem due to its high rehospitalization rate and mortality (1–3). It is well known that the occurrence and development of heart failure are mainly related to ventricular remodeling, including hypertrophy of cardiomyocytes, myocardial fibroblast proliferation, and myocardial fibrosis. These changes are inseparable from the inflammatory response, and it has been reported that the activation and infiltration of immune cells in the myocardium are directly involved in the pathogenesis of heart failure (4, 5). It is worth noting that with heart failure, a large number of T lymphocytes accumulate in myocardial tissue, and their ability to express inflammatory factors is significantly enhanced (6), which leads to a sustained inflammatory response and the progression of myocardial remodeling (7). Nevertheless, the immune regulatory mechanism of T lymphocytes involved in myocardial inflammation in ADHF is still unclear.

T cell immunoglobulin and mucin domain-containing protein 3 (Tim-3, encoded by hepatitis A virus-cellular receptor 2 [*HAVCR2*]) is a unique inhibitory co-receptor expressed restrictedly on the surface of immune cells and mediates immune tolerance by regulating the activity of immune cells like T cells (8). In recent years, numerous studies have examined the negative regulatory role of Tim-3-mediated immune responses in a variety of diseases (9–13). However, some scholars found that Tim-3 can convert to inflammatory and tissue injury phenotypes under acute stimulation (14–16). Currently, new advances have been made in the field of Tim-3-mediated regulation of immune inflammation in heart failure; Yu et al. (17) discovered that the proportion of Tim-3 on CD4^+^ and CD8^+^ T cells was significantly increased in patients with chronic heart failure, suggesting that Tim-3 may induce T cell dysfunction in patients with chronic heart failure, participate in the process of myocardial remodeling, and accelerate heart failure progression. Despite that, to our knowledge, the relationship between Tim-3 expression and ADHF remains unclear.

Consequently, we speculated that Tim-3 may be involved in the process of myocardial remodeling in ADHF by upregulating the activity and proliferation of CD4^+^ and CD8^+^ T cells, and affects the prognosis of ADHF patients. To test this hypothesis, we analyzed the characteristics of Tim-3 expression on peripheral CD4^+^ and CD8^+^ T cells in patients with ADHF and assessed its predictive value for major adverse cardiovascular and cerebrovascular events (MACCE) within 12 months after ADHF.

## Materials and methods

### Study population and design

From December 2020 to February 2021, 84 consecutive patients with ADHF admitted to the Department of Cardiology, Affiliated Sixth People’s Hospital, Shanghai Jiao Tong University School of Medicine were enrolled as the ADHF group. Inclusion criteria were as follows: patients with ADHF diagnosed according to the 2016 European Society of Cardiology guidelines for the diagnosis and treatment of acute heart failure and defined as rapid or chronic onset of decompensated HF or decompensation of chronic HF, with signs and symptoms of HF resulting in unplanned hospitalization (18). 83 patients with other diseases were also recruited as the non-HF group. The exclusion criteria were as follows: inflammatory diseases, autoimmune diseases, active infections or malignant tumors, and clinical data being incomplete.

Peripheral blood was collected from the patients on the day of admission. Peripheral blood mononuclear cells were isolated. The expression of Tim-3 on CD4^+^ and CD8^+^ T cells were detected by flow cytometry. Other biochemical indicators including brain natriuretic peptide (BNP), N-terminal pro-brain natriuretic peptide (NT-proBNP), serum creatinine (Scr), cardiac-specific enzymes like creatine kinase-MB (CK-MB), cardiac troponin I (cTnI) and inflammatory indicators were also evaluated. All patients eligible for coronary heart disease underwent coronary angiography. Coronary angiography was performed by standard techniques, and significant coronary artery disease was visually diagnosed if there was ≥50% diameter stenosis in the major epicardial coronary arteries. Left main disease was counted as two-vessel disease, and the presence of more than two significant coronary artery lesions was considered multi-vessel disease.

Echocardiographic assessment was performed within 24 hours of admission. We measured echocardiographic parameters according to the current guidelines of the American Society of Echocardiography (19). Left atrial diameter and left ventricular end diastolic diameter (LVEDD) were routinely evaluated by two-dimensional ultrasound, and left ventricular ejection fraction (LVEF) was measured by modified Simpson method.

Each patient’s baseline clinical data, biochemical and angiographic variables, and ultrasonic cardiogram results were recorded. All patients with acute heart failure were followed up by a cardiovascular physician.

This study was approved by the Ethics Committee of Shanghai Jiao Tong University Affiliated Sixth People’s Hospital. All patients included in the study volunteered to participate in this clinical study and signed an informed consent form.

### Peripheral blood mononuclear cells isolation

Peripheral blood was collected in tubes containing sodium heparin (BD Biosciences, NJ, USA) by venipuncture. Human peripheral blood mononuclear cells (PBMCs) were isolated from peripheral blood samples via density gradient centrifugation using Lymphoprep Solution (Axis-Shield, Dundee, Scotland). PBMCs were washed with 2% fetal bovine serum in cold phosphate-buffered saline and frozen in cryopreservation medium (20% fetal bovine serum and 10% dimethyl sulfoxide in Dulbecco’s Modified Eagle Medium) until further use.

### Flow cytometry

**FIGURE 1:**
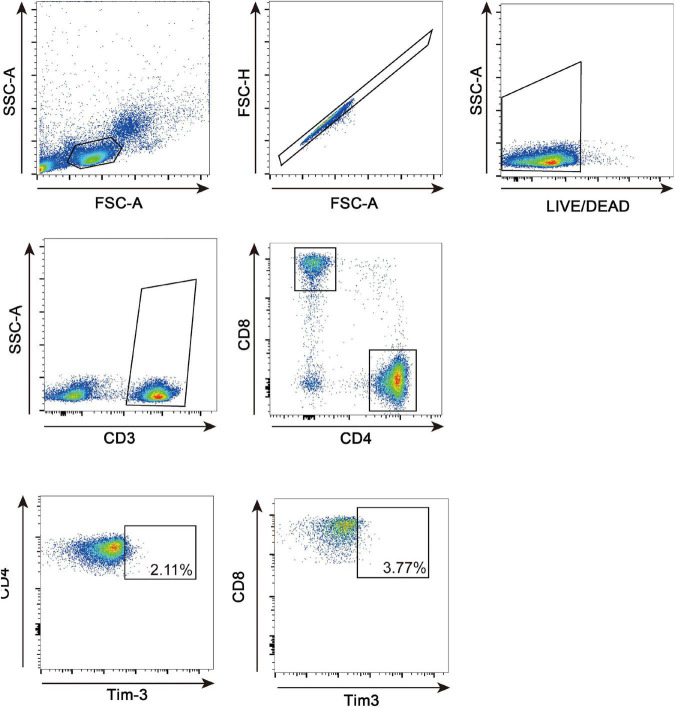
FACS gating strategy for lymphocytes. Representative flow cytometric dot plots to determine CD4^+^ and CD8^+^ lymphocytes in human PBMCs. PBMC were gated as lymphocytes on forward and side scatter density plots. Then, singlets were gated on FSC-A and FSC-H plots, and live cells were gated for subsequent analysis. CD4^+^ and CD8^+^ T cells were identified from the positive population for CD3. CD4^+^ Tim-3^+^ and CD8^+^Tim-3^+^ subpopulation were gated on CD4^+^ and CD8^+^ T cells, respectively.

PBMCs (2 × 10^6^/tube) were stained for 15 min on ice using Zombie NIR Fixable Viability Kit (Biolegend, CA, USA) to gate out dead cells. The cells were washed once, incubated for 30 min on ice with a cocktail composed of the following antibodies: anti-CD3 FITC antibody (UCHT1, Biolegend), anti-CD4 PerCP/Cyanine 5.5 antibody (OKT4, Biolegend), anti-CD8a APC antibody (RPA-T8, Biolegend) and anti-CD366 PE antibody (F38-2E2, Biolegend). To prepare Full Minus One (FMO) control, we added all other antibodies and Zombie NIR Fixable Viability dye except anti-CD366 PE antibody into each sample and stained in the same condition. Labeled cells were washed once with staining buffer and analyzed by BD Cantoplus Analyzer (BD Biosciences, NJ, USA) and FlowJo software (BD Biosciences, NJ, USA). Lymphocytes were selected based on FSC-A vs. side scatter area. We analyzed forward scatter area vs. forward scatter height to remove doublets. The analysis strategy for evaluating the proportion of Tim-3 on CD4^+^ and CD8^+^ T cells is presented in Figure 1. The precise gating bound of Tim-3 in each sample was determined according to corresponding Full Minus One control (Supplementary Figure 1).

### Follow-up

Clinical follow-up data were obtained from the patient’s inpatient medical records, regular outpatient visits, and telephone interviews. 84 patients with ADHF were followed up for 12 months. The primary clinical endpoint was MACCE during follow-up (12 months from ADHF). MACCE were defined as rehospitalization for heart failure, unplanned coronary lesion revascularization, acute coronary syndrome at follow-up, ischemic cerebrovascular accident, and cardiac death within 12 months. All potential endpoint events were adjudicated by an assessment committee whose members were blind to patient characteristics.

### Statistical analysis

Continuous variables are presented as mean ± standard deviation (SD) for normally distributed variables, otherwise, median (25th–75th percentile) for non-normally distributed variables. Categorical parameters, such as enumeration data, are expressed as percentages (%). Comparisons of continuous variables were performed by two-sample Student’s *t*-test or Mann-Whitey-U test (for significantly skewed variables). The chi-square test was used to assess differences in categorical data. Logistic multivariate regression analysis was performed for indicators with *p* < 0.05 in univariate analysis to calculate odds ratio (OR) and 95% confidence interval (95%Cl). Spearman correlation analysis was used to calculate correlation coefficient. The area under the receiver operating characteristic curve (AUC) was used to analyze the prediction of Tim-3 for the risk of ADHF; the effect of Tim-3 expression on CD4^+^ and CD8^+^ T cells on the incidence of MACCE within 12 months after ADHF was evaluated with Cox regression analysis, and expressed as hazard ratio (HR) and respective 95% confidence interval (CI) for each standard deviation (1-SD) increase in continuous variables, and adjusted for potential confounders in a multivariate model. In this analysis, high Tim-3 expression on CD4^+^ and CD8^+^ T cells was defined as the mean Tim-3 expression within the 2 highest quartiles (Q3-Q4), whereas low Tim-3 expression on CD4^+^ and CD8^+^ T cells was defined as the first to second quartile (Q1–Q2), and stratified by high (Q3–Q4) and low (Q1–Q2) expression of Tim-3 on CD4^+^ and CD8^+^ T cells. Kaplan-Meier survival curves were used to estimate the survival status of patients with high and low Tim-3 expression on CD4^+^ and CD8^+^ T cells within 12 months after ADHF and compared using the log-rank test. Statistical significance was defined as *p* < 0.05. All analyses were performed using IBM SPSS software (version 22.0 for Windows; SPSS, Inc., Chicago, IL, USA). The ROC curve and Kaplan-Meier survival curves were plotted using GraghPadPrism7.0.

## Results

### Baseline characteristics

Eighty-four patients with ADHF and 83 patients without HF were enrolled in this study. The baseline clinical data, biochemical and angiographic variables, and echocardiographic parameter results are shown in Table 1.

**TABLE 1:** Clinical characteristics of patients.

Parameters	Overall (*n* = 167)	ADHF group (*n* = 84)	Non-HF group (*n* = 83)	*P*-value
Age (years)	66 (54–73)	69 (55–82)	61 (54–69)	0.001
Male, *n* (%)	112 (67.10%)	60 (71.40%)	52 (62.70%)	0.228
Hypertension, *n* (%)	78 (46.70%)	45 (53.60%)	33 (39.80%)	0.074
Diabetes, *n* (%)	36 (21.60%)	22 (26.20%)	14 (16.90%)	0.143
Atrial fibrillation, *n* (%)	23 (13.80%)	19 (22.60%)	4 (4.80%)	0.001
DCM, *n* (%)	16 (9.60%)	16 (19.00%)	0 (0)	–
CHD, *n* (%)	60 (35.90%)	39 (46.40%)	21 (25.30%)	0.004
AMI, *n* (%)	32 (19.20%)	32 (38.10%)	0 (0)	–
Target vessel				
LAD, *n* (%)	52 (31.10%)	33 (39.30%)	19 (22.90%)	0.022
LCX, *n* (%)	24 (14.40%)	17 (20.20%)	7 (8.40%)	0.030
RCA, *n* (%)	24 (14.40%)	14 (16.70%)	10 (12.00%)	0.395
Single-vessel disease, *n* (%)	32 (19.20%)	17 (20.20%)	15 (18.10%)	0.722
Multi-vessel disease, *n* (%)	31 (18.60%)	23 (27.40%)	8 (9.60%)	0.003
LA (mm)	39.20±6.65	42.21±6.93	35.75±4.22	<0.001
LVEDD (mm)	49.96±7.30	53.23±7.95	46.19±3.95	<0.001
LVEF (%)	59 (46.0–64.0)	47 (36.5–55.5)	64 (62.0–67.0)	<0.001
BNP (pg/mL)	125.5 (29.0–1254.8)	1,160 (472.5–1931.5)	29 (17.0–44.0)	<0.001
NT-proBNP (pg/mL)	184 (59.4–5090)	5,090 (2660.0–14200.0)	59.4 (39.2–85.8)	<0.001
D-Dimer (mg/L)	0.41 (0.22–1.04)	0.86 (0.41–1.91)	0.24 (0.15–0.41)	<0.001
Lac (mmol/L)	1.80 (1.35–2.85)	1.80 (1.40–2.90)	1.50 (0.60–2.40)	0.437
Scr (μmol/L)	81.5 (66.8–110.5)	95.5 (73.0–126.0)	71.0 (62.8–85.3)	<0.001
cTnI (μg/L)	0.02 (0.004–0.335)	0.34 (0.04–7.98)	0.005 (0.003–0.006)	<0.001
CRP (mg/L)	5.03 (0.5–12.85)	10.91 (4.59–31.49)	0.5 (0.49–5.55)	<0.001
ESR (mm/h)	8.0 (2.0–19.0)	17.0 (6.0–33.0)	4.0 (2.0–11.0)	<0.001
PCT (ng/mL)	0.66 (0.04–0.16)	0.07 (0.04–0.61)	0.06 (0.03–0.09)	0.305
CD4^+^ T cells (%)	61.6 (52.1–78.7)	63.7 (53.8–84.3)	59.3 (43.5–70.8)	0.015
CD8^+^ T cells (%)	19.6 (6.4–33.6)	26.9 (15.3–37.8)	11.5 (4.0–31.4)	<0.001

DCM, Dilated cardiomyopathy; CHD, Coronary heart disease; AMI, Acute myocardial infarction; LAD, Left anterior descending branch; LCX, Left circumflex branch; RCA, Right coronary artery; LA, Left atrium; LVEDD, End diastolic diameter of left ventricle; LVEF, Left ventricular ejection fraction; BNP, Brain natriuretic peptide; NT-proBNP, N-terminal pro-brain natriuretic peptide; Lac, Lactic acid; Scr, Serum creatinine; cTnI, cardiac Troponin I; CRP, C-reactive protein; ESR, Erythrocyte sedimentation rate; PCT, Procalcitonin.

We dichotomized patients into two groups, the ADHF and non-HF group. Compared with those in the non-HF group, the patients in the ADHF group were older [69 years (55–82 years) vs. 61 years (54–69 years), *p* = 0.001] and had no gender difference (71.4 vs. 62.7%, *p* = 0.228). The initial-presented levels of BNP and NT-proBNP, as well-known markers of ADHF, were significantly different between the two groups (*p* < 0.001). The angiographic results suggested that the ADHF group had considerably more multi-vessel disease and anterior descending branch (LAD) coronary lesions than the non-HF group had. In echocardiographic parameters, the baseline LVEF of the ADHF group was lower than those of the non-HF group [47% (36.5–55.5%) vs. 64% (62.0–67.0%), *p* < 0.001], and the levels of LVEDD were significantly higher in the HF group than in the non-HF group (53.2 ± 7.9 mm vs. 46.2 ± 3.9 mm, *p* < 0.001). Intriguingly, the values of Scr [95.5 μmol/L (73.0–126.0 μmol/L) vs. 71.0 μmol/L (62.8 μmol/L −85.3 μmol/L), *p* < 0.001] tended to be higher in patients with ADHF. Notably, we found that the frequencies of both CD4^+^ (*p* = 0.015) and CD8^+^ (*p* < 0.001) T cells were significantly increased in the ADHF group compared with the non-HF group (Table 1).

### Tim-3 levels in the acute decompensated heart failure and non-HF groups

**FIGURE 2:**
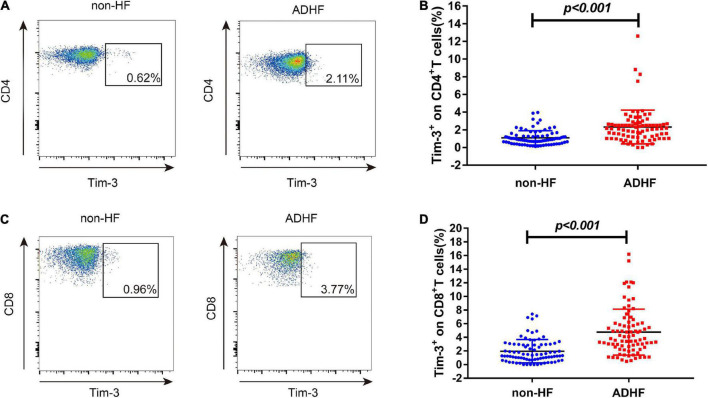
The expression of Tim-3 on CD4^+^ T cells and CD8^+^ T cells in non-HF and ADHF groups. Peripheral blood mononuclear cells (PBMCs) were isolated from ADHF group (*n* = 84) and the non-HF group (*n* = 83). **(A)** Flow cytometry analysis of Tim-3 expression on CD4^+^ T cells. **(B)** The statistical graph of Tim-3 expression on CD4^+^ T cells is shown for ADHF group [*n* = 84, 2.08% (1.15–2.67%)] and non-HF group [*n* = 83, 0.88% (0.56–1.39%)]. **(C)** Flow cytometry analysis of Tim-3 expression on CD8^+^ T cells. **(D)** The statistical graph of Tim-3 expression on CD8^+^ T cells is shown for ADHF group [*n* = 84, 3.81% (2.24–6.03%)] and non-HF group [*n* = 83, 1.36% (0.76–3.00%)].

We analyzed the expression of Tim-3 on CD4^+^ and CD8^+^ T cells in both ADHF and non-HF group by flow cytometry (Figures 2A,C). As shown in Figure 2B, we observed that the expression of Tim-3 on CD4^+^T cells was increased in the ADHF group [2.08% (1.15–2.67%) vs. 0.88% (0.56–1.39%), *p* < 0.001] than those in the non-HF group. Similarly, the expression of Tim-3 on CD8^+^ T cells showed a tendency to be higher than those in the non-HF group [3.81% (2.24–6.03%) vs. 1.36% (0.76–3.00%), *p* < 0.001; Figure 2D].

### Correlation between Tim-3 expression and different heart failure indexes

As illustrated in Table 2, the expression of Tim-3 on CD4^+^ T cells was positively correlated with NT-proBNP (ρ = 0.426, *p* < 0.001) and BNP (ρ = 0.428, *p* < 0.001), whereas it was negatively correlated with LVEF (ρ = -0.370, *p* < 0.001). Similarly, the expression of Tim-3 on CD8^+^ T cells was positively correlated with NT-proBNP (ρ = 0.423, *p* < 0.001); BNP (ρ = 0.362, *p* < 0.001) and LVEDD (ρ = 0.250, *p* = 0.003), and negatively correlated with LVEF (ρ = -0.377, *p* < 0.001).

**TABLE 2:** Correlation between Tim-3 expression and different heart failure indexes.

Variable		Tim-3^+^ on CD4^+^ T cells(%)	Tim-3^+^ on CD8^+^ T cells(%)
NT-proBNP (pg/mL)	ρ	0.426	0.423
	*p*	<0.001	<0.001
BNP (pg/mL)	ρ	0.428	0.362
	*p*	<0.001	<0.001
LVEDD (mm)	ρ	0.157	0.250
	*p*	0.061	0.003
LVEF (%)	ρ	−0.370	−0.377
	*p*	<0.001	<0.001

Spearman’s coefficient of rank correlation was used in calculation. BNP, Brain natriuretic peptide; NT-proBNP, N-terminal pro-brain natriuretic peptide; LVEDD, End diastolic diameter of left ventricle; LVEF, Left ventricular ejection fraction; ρ, correlation coefficient; p, calculated probability.

### Logistic regression analysis of specific markers as risk factors for the assessment of acute decompensated heart failure

As Table 3 indicates, univariate analysis showed that the high Tim-3 expression on CD4^+^ and CD8^+^T cells were associated with ADHF events (OR 3.72; 95% CI: 1.96–7.06, *p* < 0.001; OR 3.35; 95% CI: 1.78–6.32, *p* < 0.001, respectively). Interestingly, advanced age (OR 1.03; 95% CI: 1.01–1.06, *p* = 0.002), coronary artery disease (OR 2.56: 95% CI: 1.33–4.93, *p* = 0.005), LAD lesions (OR 2.18; 95% CI: 1.11–4.28, *p* = 0.023), and multi-vessel coronary lesions (OR 3.54; 95% CI: 1.48–8.46, *p* = 0.005) were also associated with ADHF events.

**TABLE 3:** Logistic regression analysis of the correlation between specific markers, risk factors and ADHF events.

Variables	Univariate analysis		Multivariate analysis	
	OR(95% CI)	*P*-value	OR(95% CI)	*P*-value
Male	1.49 (0.78–2.85)	0.229	–	–
Age	1.03 (1.01–1.06)	0.002	1.04 (1.01-1.06)	0.002
Hypertension	1.75 (0.95–3.23)	0.075	–	–
Diabetes	1.75 (0.82–3.71)	0.146	–	–
CHD	2.56 (1.33–4.93)	0.005	2.56 (0.62-10.64)	0.197
Target vessel				
LAD	2.18 (1.11–4.28)	0.023	0.85 (0.19-3.88)	0.833
LCX	2.76 (1.08–7.05)	0.135	–	–
RCA	1.46 (0.61–3.50)	0.397	–	–
Single-vessel disease	1.15 (0.53–2.49)	0.722	–	–
Multi-vessel disease	3.54 (1.48–8.46)	0.005	1.47 (0.45-4.82)	0.528
CD4^+^ Tim-3^+^ high level	3.72 (1.96–7.06)	<0.001	2.76 (1.34-5.65)	0.006
CD8^+^ Tim-3^+^ high level	3.35 (1.78–6.32)	<0.001	2.58 (1.26-5.31)	0.010

CHD, Coronary heart disease; LAD, Left anterior descending branch; LCX, Left circumflex branch; RCA, Right coronary artery; CD4^+^Tim-3^+^ high level, The highest quartile (Q3–Q4) of Tim-3 expression on CD4^+^T cells; CD8^+^Tim-3^+^ high level, The highest quartile (Q3–Q4) of Tim-3 expression on CD8^+^T cells.

By using multivariate logistic regression analysis, after adjustment for potential confounders, we found that high level of Tim-3 expressed on CD4^+^ and CD8^+^T cells remained independent predictors of ADHF (OR 2.76; 95% CI: 1.34–5.65, *p* = 0.006; OR 2.58; 95% CI: 1.26–5.31, *p* = 0.010, respectively).

### Predictive performances of Tim-3 expressed on CD4^+^ and CD8^+^ T cells for acute decompensated heart failure

**FIGURE 3:**
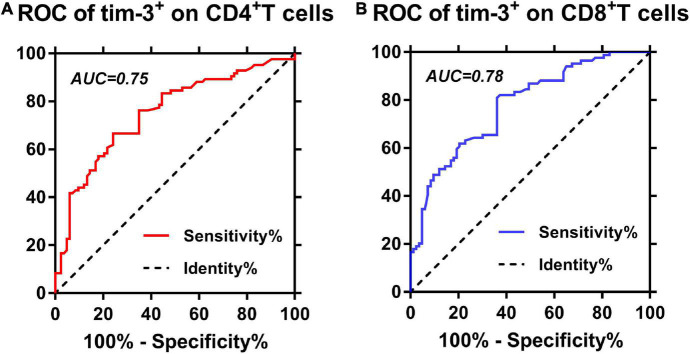
Predictive performances of Tim-3 expression on CD4^+^ and CD8^+^ T cells. **(A)** ROC curves for Tim-3 expression on CD4^+^ T cells. **(B)** ROC curves for Tim-3 expression on CD8^+^ T cells.

We further investigated the predictive performance and optimal cutoff value of Tim-3 expression for ADHF events by ROC curve analysis. As reflected in Figure 3 and Table 4, the ROC curve analysis showed that AUC for Tim-3 expressed on CD4^+^ T cells was 0.75 (95% CI: 0.68–0.83), with a sensitivity of 71.3% and a specificity of 71.3% at the optimal cutoff value of 1.400%. The positive likelihood ratio (PLR) and negative likelihood ratio (NLR) values were 2.48 and 0.40, respectively. The AUC for Tim-3 expression on CD8^+^ T cells was 0.78 (95% CI: 0.71–0.85) with a sensitivity of 72.5% and a specificity of 72.3% at the cutoff value of 1.965%. The PLR and NLR values were 2.62 and 0.38, respectively.

**TABLE 4:** Predictive values of each biomarker.

Makers	AUC (95% CI)	Cut-off	Sensitivity	Specificity	PPV	NPV	PLR	NLR
Tim-3^+^ on CD4^+^ cells	0.75 (0.68, 0.83)	1.400	71.3%	71.3%	73.7%	69.2%	2.48	0.40
Tim-3^+^ on CD8^+^ cells	0.78 (0.71, 0.85)	1.965	72.50%	72.3%	69.4%	76.8%	2.62	0.38

PPV, Positive predictive value; NPV, Negative predictive value; PLR, Positive likelihood radio; NLR, Negative likelihood ratio.

### Survival analysis

#### Kaplan-Meier survival curves

**FIGURE 4:**
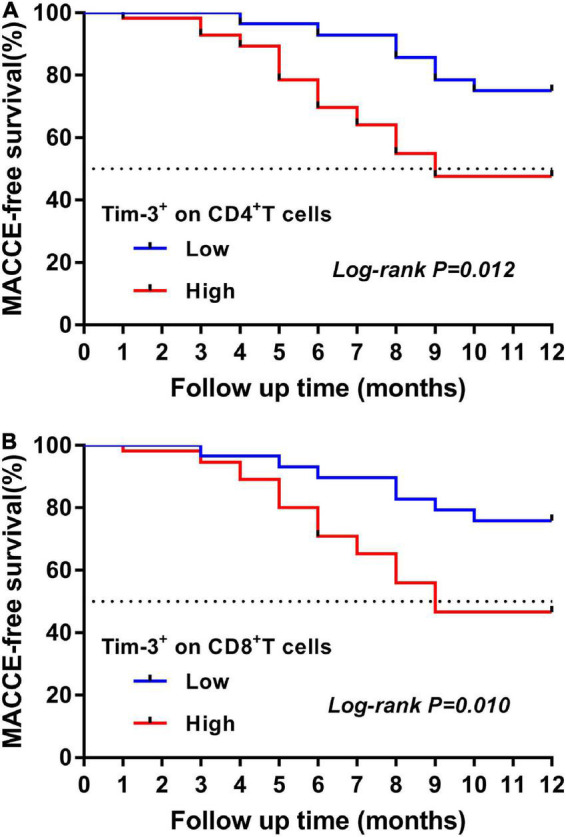
Kaplan-Meier curve for the 12-month MACCE-free survival in patients with ADHF. **(A)** Time-to-event curves for the highest quartile (Q3–Q4) of Tim-3 expression on CD4^+^ T cells vs. the lowest quartiles (Q1–Q2) measured in ADHF patients according to the primary endpoints. The log rank test was used to compare the survival curves. **(B)** Time-to-event curves for the highest quartile (Q3–Q4) of Tim-3 expression on CD8^+^ T cells vs. the lowest quartiles (Q1–Q2) measured in ADHF patients according to the primary endpoints. The log rank test was used to compare the survival curves.

After a median follow-up of 12 months in patients with ADHF, a total of 37 (44%) patients had MACCE (5 cardiac deaths, 22 readmissions due to HF, and 10 readmissions due to acute coronary syndrome). Patients with ADHF were divided into high (Q3–Q4) and low (Q1–Q2) expression of Tim-3 on CD4^+^ and CD8^+^T cells groups according to the quartiles. The Kaplan-Meier curve depicted the follow-up without MACCE (MACCE-free) survival by comparing high and low expression of Tim-3 on CD4^+^ and CD8^+^ T cells in ADHF patients, followed by comparison of survival curves using log-rank test. As presented in Figure 4, Kaplan-Meier survival curves showed that ADHF patients with higher Tim-3 expression on CD4^+^ T cells (*p* = 0.007; Figure 4A) and CD8^+^ T cells (*p* = 0.010; Figure 4B) had shorter survival times without MACCE.

#### Univariate and multivariate cox proportional hazard regression analyses for the risk of major adverse cardiac and cerebrovascular events within 12 months after acute decompensated heart failure

According to Table 5, the incidence of MACCE within 12 months was higher in patients with LVEF < 50% (HR: 3.936; 95% CI:1.71–9.07; *p* = 0.001) and LVEDD > 50 mm (HR: 2.531; 95% CI: 1.18–5.43; *p* = 0.017). Analysis of individuals presenting with DCM showed an association with MACCE (HR: 2.968; 95% CI: 1.48–5.97; *p* = 0.002). Noteworthy, high expression of Tim-3 on CD4^+^ T cells demonstrated a strong association with MACCE in the entire study cohort with a crude HR per 1-SD of 2.685 (95% CI: 1.17–6.14, *p* = 0.019). In addition, high expression of Tim-3 on CD8^+^ T cells correlated strongly with MACCE with an HR per 1-SD of 2.730 (95% CI: 1.19–6.24, *p* = 0.017). After adjustment for confounders, high Tim-3 expression on CD4^+^ and CD8^+^ T cells were independent predictors of MACCE at 12 months after ADHF (HR: 2.613, 95% CI: 1.11–6.13, *p* = 0.027; HR: 2.762, 95% CI: 1.15–6.63, *p* = 0.023; respectively).

**TABLE 5:** Univariate and multivariate cox proportional hazard regression analyses for the risk of MACCE within 12 months among patients with ADHF.

Variables	Univariate analysis			Multivariate analysis		
	HR	95% CI	*P*	HR	95% CI	*P*
Age	0.986	0.97–1.00	0.171	–	–	–
Male	0.908	0.44–1.89	0.796	–	–	–
Hypertension	0.920	0.48–1.77	0.803	–	–	–
Diabetes	1.010	0.49–2.10	0.979	–	–	–
CHD	0.953	0.49–1.84	0.886	–	–	–
Target vessel						
LAD	1.051	0.54–2.05	0.885	–	–	–
Multi-vessel disease	1.327	0.65–2.70	0.435	–	–	–
Atrial fibrillation	1.038	0.49–2.21	0.923	–	–	–
DCM	2.968	1.48–5.97	0.002	1.443	0.64–3.24	0.374
LVEDD > 50 mm	2.531	1.18–5.43	0.017	1.213	0.47–3.11	0.688
LVEF < 50%	3.936	1.71–9.07	0.001	3.873	1.44–10.42	0.007
BNP > 500 pg/mL	1.638	1.79–3.41	0.187	–	–	–
NT-proBNP > 1,200 pg/mL	3.410	0.56–20.76	0.183	–	–	–
CD4^+^ Tim-3^+^ high level	2.685	1.17–6.14	0.019	2.613	1.11–6.13	0.027
CD8^+^ Tim-3^+^ high level	2.730	1.19–6.24	0.017	2.762	1.15–6.63	0.023

DCM, Dilated cardiomyopathy; CHD, Coronary heart disease; LAD, Left anterior descending branch; LVEDD, End diastolic diameter of left ventricle; LVEF, Left ventricular ejection fraction; CD4^+^ Tim-3^+^ high level, The highest quartile (Q3–Q4) of Tim-3 expression on CD4^+^T cells; CD8^+^Tim-3^+^ high level, The highest quartile (Q3–Q4) of Tim-3 expression on CD8^+^T cells.

## Discussion

In the present study, we demonstrated that Tim-3 was up-regulated on both CD4^+^ and CD8^+^ T cells in patients with ADHF, and its expression correlated positively with NT-proBNP levels and BNP levels. High expression of Tim-3 on CD4^+^ and CD8^+^ T cells were independent predictors of ADHF events. COX analysis suggested that the up-regulation of Tim-3 expression on CD4^+^ and CD8^+^ T cells was significantly associated with MACCE events within 12 months after ADHF.

Over recent years, scholars have found that the immune inflammatory response plays an important role in ventricular remodeling. Epelman et al. (20) proposed that inflammatory cytokines were up-regulated in cardiac tissue of heart failure model cells. Immune regulatory receptors on the surface of inflammatory cells also regulate left ventricular hypertrophy (21). The current experimental results in animal models show that CD4^+^ T cells accumulated in the left ventricular tissue of mice with congestive heart failure (22) and participate in collagen production through proliferation and activation (5). Under cardiac pressure overload, dendritic cells induced CD4^+^ T cell proliferation by accumulating immunoregulatory signaling proteins (23), and on the other hand, the involvement of chemokines promotes the activation of CD4^+^ T cells and cardiac infiltration, promote myocardial fibrosis and left ventricular dysfunction (24). Notably, recent studies have revealed the relationship between specific subpopulation of CD8^+^ T cells and myocardial remodeling. In the early stage of heart failure, the frequency of CD8^+^ T cells in the myocardium is significantly increased (25). The dynamic interaction between myocardial infiltrating CD8^+^ T cells and macrophages promotes myocardial hypertrophy and plays an important role in the occurrence of adaptive myocardial remodeling (26). In our study, the frequency of CD4^+^ and CD8^+^ T cells in ADHF group was significantly increased. Our data suggest that CD4^+^ and CD8^+^ T cells under myocardial overload in ADHF patients may be influenced by immune regulation to proliferate and activate participating in myocardial fibrosis and heart failure progression.

Tim-3 is an immunomodulatory and tolerogenic regulator that is expressed in innate and adaptive immune cells. Accumulating evidence suggests that abnormal expression of Tim-3 on peripheral CD4^+^ and CD8^+^ T cells is closely associated with autoimmune diseases, viral infections, and cancers. It has been previously reported that Tim-3 often acts as a negative regulator to mediate T cell exhaustion (27). The results of studies published so far mostly support the conclusion that Tim-3 inhibits T cell responses, especially when chronic stimuli are involved (28–30). In contrast, several reports provided evidence that Tim-3 could promote both T-lymphocyte proliferation and proinflammatory cytokine production under acute stimulation (14–16). These results suggest that the immunomodulatory function of Tim-3 may be reversed in the presence of its different ligands. In our data, ADHF patients exhibit an increased expression of Tim-3 on CD4^+^ and CD8^+^ T cells. It depicts that the up-regulation of Tim-3 expression on CD4^+^ and CD8^+^ T cells in ADHF patients may increase the number of specific subpopulations of CD4^+^ and CD8^+^ T cells. Notably, we show that the high expression of Tim-3 on CD4^+^ and CD8^+^T cells is an independent predictor of ADHF. Further ROC analysis revealed the expression of Tim-3 on CD4^+^ and CD8^+^T cells provides a good diagnosis of ADHF (Figure 3). Our study indicated that Tim-3 seems to be a structural positive regulator of T cell function during ADHF episodes. It can be speculated that in these patients, Tim-3 positively regulates T lymphocyte function by binding to HLA-B–associated transcript 3 (31) in response to acute myocardial injury stimulus (15). It expands the infiltration and accumulation of CD4^+^ and CD8^+^ T cells in the myocardium and promotes the release of myocarditis factors (such as IL-2, IL-6, etc.). Also, it induces the production of cytotoxic T cells and releases perforin and granzyme, thereby aggravating myocardial inflammatory injury and pathological hypertrophy (32). However, studies are still needed to confirm the potential biological role of Tim-3 in the development of ADHF.

Based on the relationship between T cells and acute heart failure (33), recent studies have continuously confirmed that the inflammatory response mediated by specific subpopulations of CD4^+^ and CD8^+^ T cells significantly affects the progression and prognosis of heart failure (34). Animal studies have shown that the proportion of CD4^+^ T cells and the production of activating factors are significantly increased in patients with acute heart failure, which is associated with the clinical outcome of acute heart failure (35). Clinically, it was also found that the high expression of CD4^+^ T cells in patients with heart failure is a strong predictor of all-cause and cardiovascular mortality in patients with heart failure and a potential marker for the progression of heart failure (36). Moreover, a significant increase in the proportion of CD8^+^ T cells was associated with deteriorating cardiac function and long-term MACCE (37, 38). Therefore, it is particularly important to explore the immunomodulatory mechanisms of T cells by specific immunomodulatory factors in ADHF patients. Notably, Tim-3 influenced the development of most diseases through immunomodulatory functions (9, 34, 39, 40). Our data show that ADHF patients with high expression of Tim-3 on CD4^+^ and CD8^+^ T cells had a higher incidence of MACCE by Kaplan-Meier analysis (Figure 4). In particular, the predictive performance of high expression of Tim-3 on CD4^+^ and CD8^+^ T cells remained in multivariate Cox proportional hazards regression analysis after adjusting for potential confounders (Table 5). Our study indicates that high co-expression of Tim-3 on CD4^+^ and CD8^+^ T cells is an independent predictor of the occurrence of MACCE in patients with ADHF, and its upregulation is associated with poor survival in ADHF patients. It has the potential utility to help identify patients at high risk of MACCE early.

## Study limitations

Our study has the following limitations. First, the number of samples included in this study is relatively small, and the expression of Tim-3 may reflect multiple diseases. To eliminate this selection bias, we excluded patients with inflammatory diseases, autoimmune diseases, active infections, or malignancies during case collection; however, potential selection bias could not be fully excluded in this study. Further large-scale prospective studies are needed to determine whether these deficiencies affect the results. Second, in this study, we only recorded Tim-3 expression of CD4^+^ and CD8^+^ T cells on the day of admission for ADHF, and no information was reported about the changes in Tim-3 expression on CD4^+^ and CD8^+^ T cells after ADHF improved with treatment and its impact on the prognosis of ADHF. Third, no antibody intervention for Tim-3 was performed in this study, and further studies are still needed to confirm the effect of immunomodulation therapy targeting Tim-3 on ADHF.

## Conclusion

The results of this study show that Tim-3 is highly expressed on both CD4^+^ and CD8^+^ T cells in ADHF, and it might be an independent predictor of MACCE incidence within 12 months after ADHF. Consequently, these findings suggest a potential immunoregulatory role of a Tim-3 signaling system in the pathogenesis of ADHF and might be used as a novel biomarker to assess the prognosis of ADHF. Targeted regulation of Tim-3 expression would be a new strategy for the treatment of ADHF, and this needs to be confirmed by further studies.

## Data availability statement

The raw data supporting the conclusions of this article will be made available by the authors, without undue reservation.

## Ethics statement

The studies involving human participants were reviewed and approved by the Independent Ethics Committee of Shanghai Sixth People’s Hospital. The patients/participants provided their written informed consent to participate in this study.

## Author contributions

XM and GX designed the research study, wrote the manuscript, performed data management, data analysis, all cell processing, and flow cytometry analysis. LZ, ZC, and CX edited and revised the article. All authors contributed to the article and approved the submitted version.

## Abbreviations

Tim-3: T cell immunoglobulin and mucin domain-containing protein 3
ADHF: Acute decompensated heart failure
MACCE: Major adverse cardiac and cerebrovascular events
HAVCR2: Hepatitis A virus-cellular receptor 2
BNP: Brain natriuretic peptide
NT-proBNP: N-terminal pro-brain natriuretic peptide
Scr: Serum creatinine
CK-MB: Cardiac-specific enzymes such as creatine kinase-MB
TnI: Troponin I
LVEDD: Left ventricular end diastolic diameter
LVEF: Left ventricular ejection fraction
PBMCs: Peripheral blood mononuclear cells
OR: Odds ratio
AUC: Area under receiver operating characteristic curve
CI: Confidence interval.
