# Supplementary material for: T cell immunoglobulin and mucin domain-containing protein 3 is highly expressed in patients with acute decompensated heart failure and predicts mid-term prognosis
Source: Front Cardiovasc Med. 2022 Sep 15;9:933532. doi: 10.3389/fcvm.2022.933532 (PMC9520239; doi:10.3389/fcvm.2022.933532)
Supplement: Supplementary file 1 [file Data_Sheet_1.PDF]

Supplementary Material

Supplementary Figures

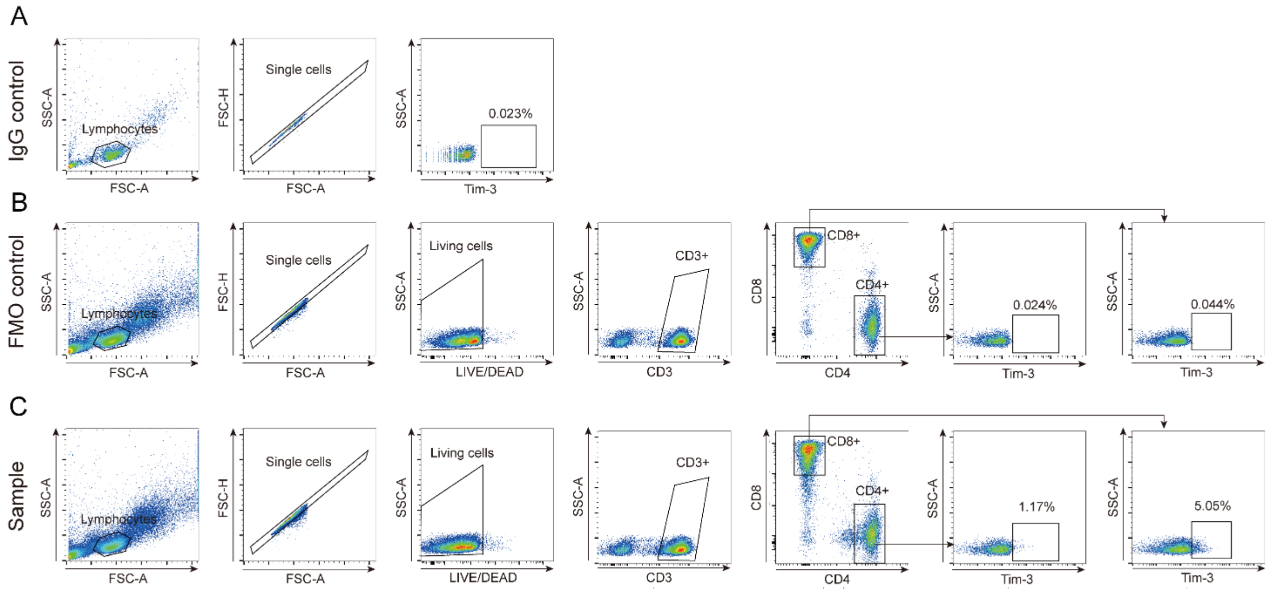

Supplementary Figure 1. The IgG control and FMO control for Tim-3.

A. The flow cytometric plots of isotype control for surface staining. B. The representative plots of Full Minus One controls for anti-Tim-3 PE antibody. C. The representative flow cytometric plots for PBMCs staining using our full flow cytometry panel.
